# Clinical and Preclinical Activity of EGFR Tyrosine Kinase Inhibitors in Non–Small-Cell Lung Cancer Harboring *BRAF* Class 3 Mutations

**DOI:** 10.1200/PO.24.00240

**Published:** 2024-12-05

**Authors:** Alessandro Di Federico, Stefania Angelicola, Mariateresa Frascino, Irene Siracusa, Beatrice Bisanti, Francesca Ruzzi, Maria Sofia Semprini, Hugo De Jonge, Andrea De Giglio, Francesca Sperandi, Stefano Brocchi, Barbara Melotti, Francesca Giunchi, Elisa Gruppioni, Annalisa Altimari, Pier-Luigi Lollini, Andrea Ardizzoni, Arianna Palladini, Francesco Gelsomino

**Affiliations:** ^1^ Medical Oncology, IRCCS Azienda Ospedaliero-Universitaria di Bologna, Bologna, Italy; ^2^ Department of Medical and Surgical Sciences (DIMEC), University of Bologna, Bologna, Italy; ^3^ Laboratory of Immunology and Biology of Metastasis, Department of Medical and Surgical Sciences (DIMEC), University of Bologna, Bologna, Italy; ^4^ Department of Molecular Medicine, University of Pavia, Pavia, Italy; ^5^ Department of Radiology, IRCCS Azienda Ospedaliero-Universitaria di Bologna, Bologna, Italy; ^6^ Pathology Unit, IRCCS Azienda Ospedaliero-Universitaria di Bologna, Bologna, Italy; ^7^ Solid Tumor Molecular Pathology Laboratory, IRCCS Azienda Ospedaliero-Universitaria di Bologna, Bologna, Italy; ^8^ IRCCS Azienda Ospedaliero-Universitaria di Bologna, Bologna, Italy; ^9^ Unità Operativa di Oncologia, Fondazione IRCCS Policlinico San Matteo, Pavia, Italy

## Abstract

**PURPOSE:**

Patients with tumors harboring *BRAF* class 3 mutations lack targeted therapies. These mutations are characterized by low/absent BRAF kinase domain activation and are believed to amplify already active RAS signaling, potentially triggered by receptor tyrosine kinases like EGFR.

**MATERIALS AND METHODS:**

Two patients with *BRAF* class 3–mutated metastatic non–small-cell lung cancer (NSCLC) were treated with erlotinib at our Institution after failure of standard therapies. Two cell lines were established from patients with *BRAF* class 3–mutated NSCLC, and their sensitivity to EGFR tyrosine kinase inhibitors (EGFR-TKIs) was assessed using *EGFR-*mutated, *BRAF* class 1 and 2–mutated, and *KRAS*-mutated NSCLC cell lines as controls.

**RESULTS:**

Patient 1, a 60-year-old male with BRAF^D594N^-mutated NSCLC, achieved complete response to erlotinib after progression on first- and second-line chemotherapy. Patient 2, a 60-year-old female with BRAF^D594G^-mutated NSCLC, achieved partial response to erlotinib after progression on first-line chemoimmunotherapy. High baseline phosphorylated EGFR values and reduced EGFR activation following erlotinib were observed in *BRAF* class 3–mutated and *EGFR*-mutated cell lines, but not in *BRAF* class 1–mutated, *BRAF* class 2–mutated, or *KRAS*-mutated lines. Erlotinib inhibited 2-dimensional growth in *BRAF* class 3–mutated cell lines (IC_50_ 6.33 and 7.11 µM) and in the *BRAF* class 2–mutated cell line (IC_50_ 5.51 µM), albeit at higher concentrations than in *EGFR*-mutated lines, whereas it showed no effect on *BRAF* class 1–mutated (IC_50_, >25 µM) or *KRAS*-mutated (IC_50_, >25 µM) lines. These findings were corroborated by 3-dimensional and sphere formation assays. In the Cancer Cell Line Encyclopedia, *BRAF* class 3–mutated NSCLC cell lines showed greater sensitivity to EGFR-TKIs compared with *BRAF* class 2–mutated and *KRAS*-mutated lines.

**CONCLUSION:**

*BRAF* class 3 mutations in NSCLC may identify a novel targetable population sensitive to EGFR-TKIs.

## INTRODUCTION

*BRAF* alterations occur in approximately 2%-5% of nonsquamous non–small-cell lung cancer (NSCLC) and can be classified into three functional classes on the basis of their effect on the BRAF kinase domain.^1,2^ Class 1, represented by BRAF^V600^ mutations, strongly activates the BRAF kinase domain as a monomer, driving the constitutive activation of the downstream MAPK pathway independently of RAS activation. Class 2 alterations are characterized by intermediate-to-high activity of the BRAF kinase domain, acting as BRAF dimers and maintaining independence from RAS for the downstream signaling process. Class 3 mutations exhibit low-to-absent activation of the BRAF kinase domain but enhance affinity with RAS, forming heterodimers with CRAF and amplifying a pre-existing RAS signal, which results in the activation of downstream pathways.^2^ Although BRAF and MEK inhibitors are effective for patients with NSCLC harboring BRAF^V600^ mutations, no targeted therapies have demonstrated convincing clinical activity for patients with BRAF^non-V600^ alterations, particularly class 3 mutations.^3-5^ In NSCLC harboring *BRAF* class 3 mutations, RAS activation by receptor tyrosine kinases like EGFR has been observed, suggesting mutant BRAF amplifies EGFR-triggered RAS signaling.^6^ Although three generations of EGFR tyrosine kinase inhibitors (EGFR-TKIs) are proven to be effective for patients with NSCLC with *EGFR* mutations, their potential against *BRAF* class 3 mutations remains unexplored.^7^ This study sought to provide the rationale and preliminary evidence of the activity of EGFR-TKIs for patients with *BRAF* class 3–mutated NSCLC.

**CONTEXT**

**Key Objective**
To assess the activity of the EGFR tyrosine kinase inhibitor (EGFR-TKI) erlotinib in *BRAF* class 3–mutated non–small-cell lung cancer (NSCLC).
**Knowledge Generated**
We report for the first time the clinical activity of the EGFR-TKI erlotinib in two patients with metastatic *BRAF* class 3–mutated NSCLC and further validated this by establishing two patient-derived cell lines sensitive to EGFR-TKIs. Erlotinib and osimertinib effectively inhibited the growth of *BRAF* class 3–mutated cell lines while showing limited to no effect on *BRAF* class 1–mutated or *KRAS*-mutated lines.
**Relevance**
*BRAF* class 3 mutations may identify patients with NSCLC who could benefit from existing targeted therapies, paving the way for clinical trials in a population currently orphan of targeted treatments.

## MATERIALS AND METHODS

### Patient Identification

We searched for patients with advanced or metastatic NSCLC harboring *BRAF* class 3 mutations without other concurring driver alterations detected by next-generation sequencing (NGS) panel (Oncomine Focus Assay; ThermoFisher Scientific, Kit RUO, Milan, Italy) who were treated with EGFR-TKIs in our institution, identifying two patients treated with erlotinib 150 mg once daily after failure of standard treatments, as per its approval based on the BR.21 study.^8^ Clinicopathologic, genomic, and outcomes data of these patients were collected by a medical oncologist (A.D.F.) through manual chart review.

The study was conducted in accordance with the Declaration of Helsinki. Human samples were collected after patients gave their informed consent. The protocol was approved by the institutional review board and by the Ethics Committee Center Emilia-Romagna Region, Italy (GR-2018-12368031). Human samples and metadata including relevant clinical data were deidentified before being shared between laboratories involved in this study. All animal procedures were performed in accordance with European directive 2010/63/UE and Italian Law (No. DL26/2014); experimental protocols were reviewed and approved by the institutional animal care and use committee of the University of Bologna and by the Italian Ministry of Health with letter 32/2020-PR.

### Establishment of Cell Lines and Patient-Derived Xenograft Models

A patient-derived xenograft (PDX) was established from a lymph node metastasis of one of the two patients with *BRAF* class 3–mutated NSCLC treated with erlotinib, harboring a BRAF^D594G^ mutation, before the administration of the EGFR-TKI, through the implantation of a tumor biopsy fragment in a BALB/c Rag2^–/–^; Il2rg^–/–^ (BRG) immunodeficient mouse.^9^ PDX-ADK-36 cell culture was derived from the tumor mass grown after the second in vivo passage. In parallel, a second cell line was established from a biopsy of a lymph node metastasis of a patient with stage IV NSCLC and a BRAF^G466V^ class 3 mutation at progression to first-line pembrolizumab (ADK-14), and a third cell line from a patient with untreated NSCLC harboring a KRAS^G12V^ mutation to serve as a control (ADK-17). The ADK-14 cell line was established and cultured in MammoCult (STEMCELL Technologies, Vancouver, Canada) supplemented with 1% fetal bovine serum (FBS; Thermo Fisher Scientific). PDX-ADK-36 and ADK-17 cell lines were established and cultured in Roswell Park Memorial Institute (RPMI) medium (Thermo Fisher Scientific) supplemented with 10% FBS. In addition, two *EGFR*-mutated (PC-9 and HCC-827, both with an E746_A750del mutation) pre-established NSCLC cell lines, one *BRAF* class 1–mutated pre-established NSCLC cell line (HCC-364, with a V600E mutation), and one *BRAF* class 2–mutated NSCLC pre-established cell line (NCI-H1395, with a G469A mutation) were used as controls. PC-9 and HCC-827 were cultured in RPMI + 10% FBS. HCC-364 and NCI-H1395 were cultured in Dulbecco's Modified Eagle Medium (DMEM) + 10% FBS; 100 U/mL penicillin and 10 µg/mL streptomycin (Thermo Fisher Scientific) were added to all mediums, and cells were grown at 37°C in a humidified atmosphere at 5% CO_2_.

### Drug Sensitivity in 2-Dimensional Culture Condition

Cells were seeded at 5,000 cells/well into a 96-well plate in MammoCult + 1% FBS (ADK-14), RPMI + 10% FBS (HCC-827, PDX-ADK-36 and ADK-17), or DMEM + 10% FBS (HCC-364, NCI-H1395). PC-9 cells were seeded at 1,000 cells/well into a 96-well plate in RPMI + 10% FBS. After 24 hours from seeding, cells were treated with drugs (all by Selleck Chemicals, Houston, TX) by adding 10 µL of a 10× solution of each drug or vehicle (for TKIs: DMSO, Merck, Milan, Italy; for cetuximab: only cell culture medium) in each well. Cell growth was assessed 72 hours later by the WST-1 cell proliferation assay (Merck) according to the manufacturer’s instructions.

### Drug Sensitivity in 3-Dimensional Culture Condition

ADK-14 and PDX-ADK-36 cells were seeded at 500 cells/well in a 24-well plate in semisolid medium—MammoCult + 1% FBS + 0.33% agar (Sea-Plaque Agarose, Lonza, Switzerland), containing drugs, with a 0.5% agarose underlay. HCC-364 and NCI-H1395 were seeded at 4,000 cells/well in a 24-well plate in semisolid medium—DMEM + 10% FBS + 0.33% agar, containing drugs, with a 0.5% agarose underlay. HCC-827 and ADK-17 cells were seeded at 2,000 cells/well and PC-9 at 500 cells/well in a 24-well plate in semisolid medium—RPMI + 10% FBS + 0.33% agar, containing drugs, with a 0.5% agarose underlay. Colonies (diameter, >90 µm) were counted 2-4 weeks later under an inverted microscope in dark field, as previously described.^10^

### Sphere Formation Assay

Cells were seeded at 10,000 cells (5,000 cells for NCI-H1395) in 4 mL complete MammoCult medium without serum in 6-well Ultra-Low adherence plate (Corning Life Sciences, Corning, NY), according to the MammoCult Human Medium Kit protocol. Drugs and vehicle were added to the medium at different doses. Cells were incubated at 37°C in a humidified 5% CO_2_ atmosphere for a week. Spheres, multicell structures with a diameter larger than 90 µm, were counted about 7 days after the seeding.^10^

### Western Blotting

Protein extraction, quantification, and Western blotting were performed as previously reported.^10^ The effect of drugs was evaluated by exposing cells to the treatment for 6 hours. Treatment was added the day after seeding. An untreated and a vehicle-treated sample ran in parallel as controls. Anti-EGFR monoclonal antibody (clone D38B1, diluted 1:1,000), anti–phospho-EGFR (Tyr1068) monoclonal antibody (clone D7A5, diluted 1:500), anti-ERK1/2 monoclonal antibody (clone 137F5, diluted 1:1,000), and anti–phospho-ERK1/2 (Thr202/Tyr204) monoclonal antibody clone (clone D13.14.4E, diluted 1:500) were used as primary antibodies. Mouse monoclonal anti-actin antibody (clone 8H10D10, 1:3,000) or anti-vinculin antibody (clone V284, 1:2,000) was used to detect reference proteins. Anti-vinculin antibody was purchased by Merck, and all the other primary antibodies were purchased from Cell Signaling Technology (Danvers, MA). Membranes were incubated with polyclonal horseradish peroxidase–conjugated anti-rabbit and anti-mouse Immunoglobulin G antibodies (Bio-Rad Laboratories, Milan, Italy). Re-Blot Plus Strong Solution (Merck) was used if needed. Proteins were detected by chemiluminescent reactions visualized using the digital imaging system Azure C600 (Azure Biosystems, Dublin, CA). Protein abundance was defined through densitometric analysis of bands by Azure Spot software (Azure Biosystems).

### Statistical Analysis

Comparisons with continuous variables were computed using the Mann-Whitney *U* test, the *t* test, or the Kruskal-Wallis test, as appropriate. All *P* values are two-sided, and confidence intervals are at the 95% level, with significance predefined to be at *P* < .05. Statistical analyses were performed using Prism GraphPad version 10 and R version 3.6.3. half maximal inhibitory concentration (IC_50_) was calculated using Prism GraphPad version 10 for the following analyses: inhibitor versus normalized response for erlotinib and osimertinib in 2-dimensional assays; inhibitor versus normalized response-variable slope for cetuximab for all cell lines except HCC-827, for which the value was calculated using the absolute IC_50_ analysis; inhibitor versus normalized response-variable slope for erlotinib in 3-dimensional (3D) and spheres. Inhibition of EGFR phosphorylation by erlotinib was performed by one-sample *t* test, and the mean of each analyzed group was compared with the hypothetical mean of 100. The number of replicates is reported in figure legends.

## RESULTS

### Antitumor Activity of Erlotinib in Two Patients With *BRAF* Class 3–Mutated NSCLC

**FIG 1.**
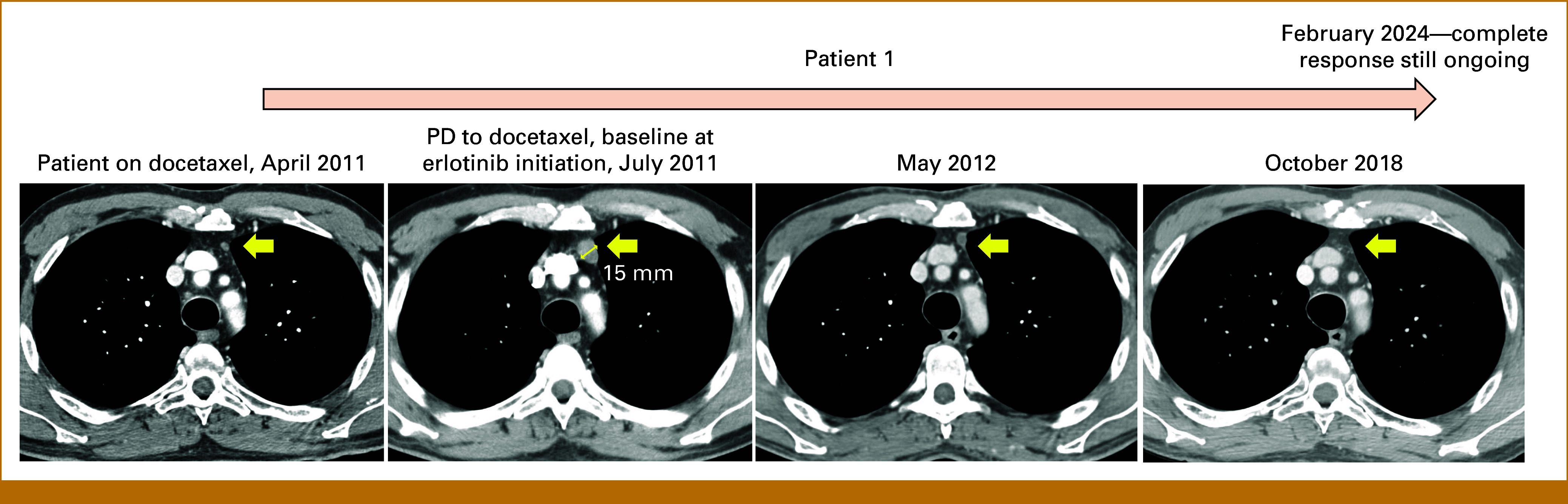
Radiographic assessment of erlotinib activity in patient 1. Computed tomography scans of patient 1 showing the tumor response to erlotinib after PD to docetaxel, with documented gradual shrinkage of a prevascular lymph node metastasis over time. PD, progressive disease.

Two patients with stage IV lung adenocarcinoma harboring a *BRAF* class 3 mutation without other concurrent genomic driver alterations were treated with erlotinib after standard treatments. Patient 1, a 60-year-old male, former smoker, was diagnosed with stage IVB lung adenocarcinoma for lymph node and brain metastases in 2010. A first-line treatment with chemotherapy (carboplatin AUC5 day 1 plus gemcitabine 1,000 mg/m^2^ days 1, 8 every 3 weeks) was administered for four cycles, and stereotactic radiation therapy was effectively performed on the single brain metastasis. Following intrathoracic nodal progressive disease (PD) at the first radiographic tumor reassessment after chemotherapy initiation, a second-line treatment with docetaxel (75 mg/m^2^ every 3 weeks) was administered for a total of 10 cycles, obtaining stable disease as best response. Disease progression was subsequently evidenced on a mediastinal lymph node, for which a third-line treatment with erlotinib 150 mg once daily was initiated in July 2011, leading to a complete response (Fig 1). NGS performed on tissue collected before the initiation of erlotinib documented a BRAF^D594N^ class 3 mutation and a CTNNB1^S37C^ mutation. The patient is still receiving erlotinib with persistent complete response after 12 years.

**FIG 2.**
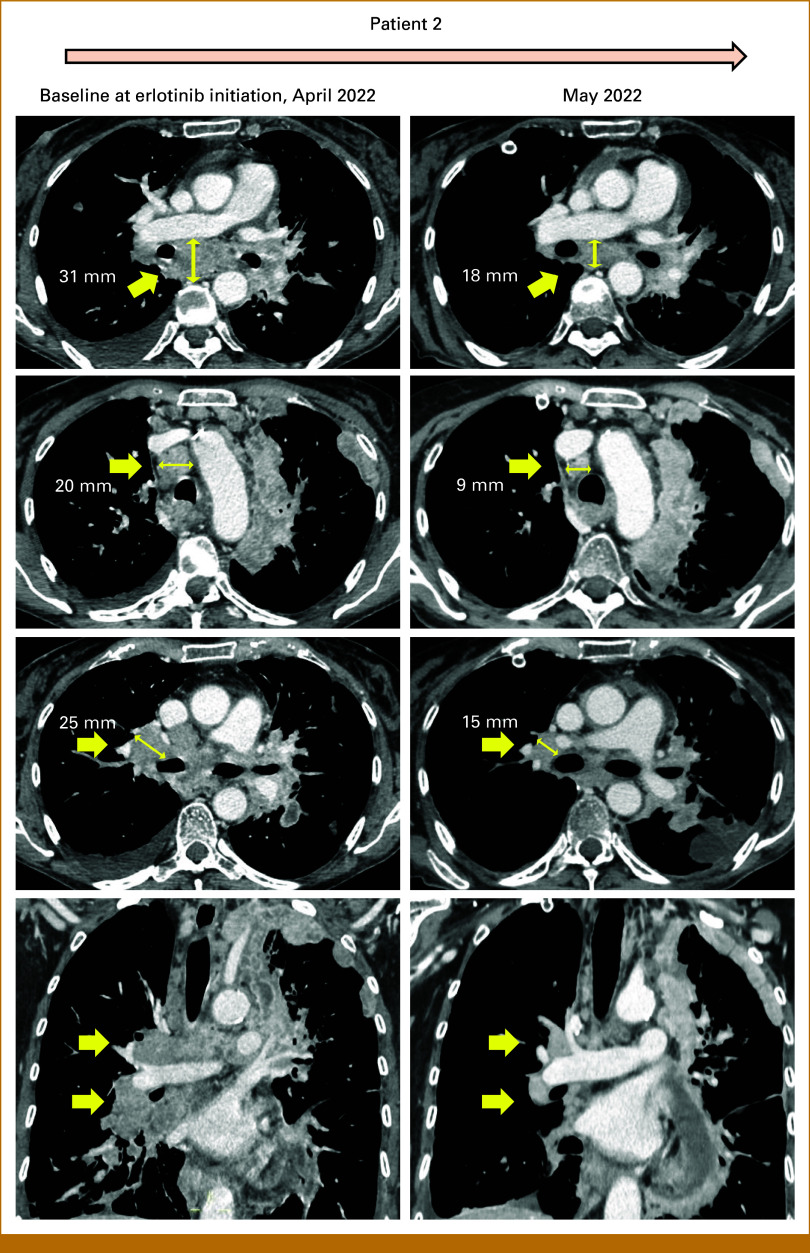
Radiographic assessment of erlotinib activity in patient 2. Computed tomography scans showing the tumor response to erlotinib in patient 2, with documented shrinkage of measurable disease in right hilar, subcarinal, and right upper paratracheal lymph node metastases.

Patient 2, a 60-year-old female, heavy smoker, was diagnosed in 2021 with stage IVB lung adenocarcinoma for pleural, bone, and lymph node metastases (Fig 2). NGS showed a BRAF^D594G^ class 3 mutation. PD-L1 tumor proportion score was 0% (clone SP263, Ventana, Roche Diagnostics, Milan, Italy). First-line chemoimmunotherapy (carboplatin AUC5, pemetrexed 500 mg/m^2^, and pembrolizumab 200 mg every 3 weeks) was administered for three cycles before clinical and radiographic evidence of PD. Given the evidence of primary treatment resistance and considering our experience with patient 1, a second-line treatment with erlotinib 150 mg once daily was started. After 1 month of treatment, computed tomography scans showed an objective partial response, with a decrease of 40% or more in measurable tumor lesions (Fig 2). Unfortunately, the patient died few days after tumor reassessment at home, likely due to an acute cardiovascular event, although the exact cause of death could not be documented.

### Activity of Erlotinib in *BRAF* Class 3–Mutated NSCLC Cell Lines

**FIG 3.**
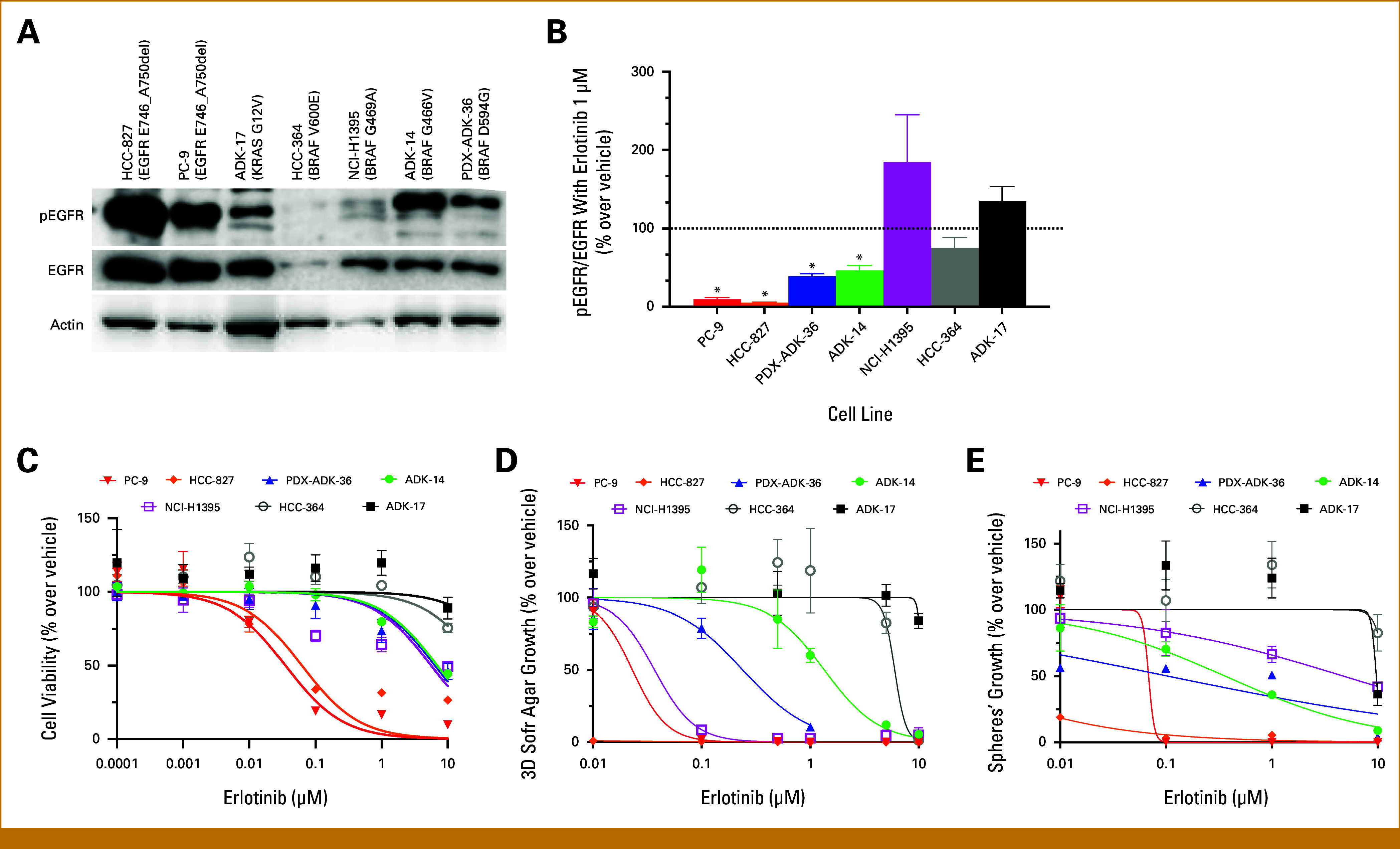
Baseline EGFR activation and comparative sensitivity of cell lines to erlotinib. (A) Western blots showing baseline EGFR activation, evaluated 30 hours after seeding. (B) Effect of erlotinib administration on EGFR phosphorylation, measured as pEGFR/EGFR ratio (n = 2-4 replicates). (C) Effect of progressively increasing doses of erlotinib (n = 2-5 experiments, each one with three replicates) on cell 2D growth. (D) Effect of progressively increasing doses of erlotinib (n = 2-4 replicates) on 3D cell growth in soft agar. (E) Effect of progressively increasing doses of erlotinib (n = 2-4 replicates) on sphere formation capability. *P < .05 over vehicle. 2D, 2-dimensional; 3D, 3-dimensional; PDX, patient-derived xenograft; pEGFR, phosphorylated EGFR.

**FIG A1.**
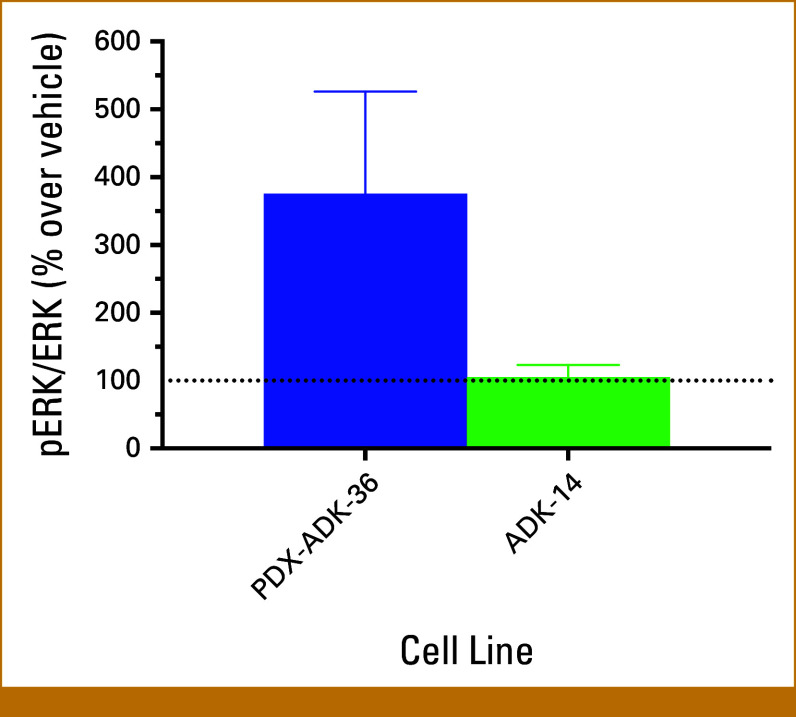
Effect of erlotinib on ERK phosphorylation in *BRAF* class 3–mutated cell lines. Effect of erlotinib treatment (1 μM) for 6 hours on ERK phosphorylation in PDX-ADK-36 and ADK-14, measured as pERK/ERK ratio. PDX, patient-derived xenograft; pEGFR, phosphorylated EGFR.

To further investigate on our clinical findings, we derived cell lines from patient 2 (PDX-ADK-36) and from another patient with NSCLC carrying a BRAF^G466V^ class 3 mutation (ADK-14). Two *EGFR*-mutated cell lines (HCC-827 and PC-9, both with the EGFR^E746_A750del^), one *BRAF* class 1–mutated cell line (HCC-364, with a BRAF^V600E^), one *BRAF* class 2–mutated cell line (NCI-H1395, with a BRAF^G469A^), and one *KRAS*-mutated cell line (ADK-17, with a KRAS^G12V^) were used as controls. Hypothesizing an overactivation of wild-type EGFR in *BRAF* class 3–mutated NSCLC cells, Western blots were performed in the seven cell lines. After 30 hours of seeding, we observed high levels of phosphorylated EGFR (pEGFR) in the two *BRAF* class 3–mutated cell lines (PDX-ADK-36 and ADK-14) and in the two *EGFR*-mutated cell lines (HCC-827 and PC-9), but not in the *KRAS-*mutated (ADK-17), *BRAF* class 1–mutated (HCC-364), and *BRAF* class 2–mutated (NCI-H1395) cell lines (Fig 3A). Consistent with our hypothesis, the first-generation EGFR-TKI erlotinib significantly reduced EGFR activation, expressed as pEGFR/EGFR ratio, in the two *BRAF* class 3–mutated cell lines (PDX-ADK-36 and ADK-14) and in the two *EGFR*-mutated (HCC-827 and PC-9) cell lines compared with the vehicle (*P* < .05), but not in the *KRAS-* (ADK-17), *BRAF* class 1–(HCC-364), and *BRAF* class 2–mutated (NCI-H1395) cell lines (Fig 3B). Notably, erlotinib did not affect the levels of phosphorylation of ERK in the two *BRAF* class 3–mutated cell lines (PDX-ADK-36 and ADK-14; Appendix Fig A1). Next, the in vitro activity of erlotinib was evaluated. Erlotinib inhibited the growth of the two *BRAF* class 3–mutated cell lines: PDX-ADK-36 (IC_50_, 6.33 µM; SE, 2.13) and ADK-14 (IC_50_, 7.11 µM; SE, 0.73; Fig 3C; Appendix Table A1). As expected, the growth of the two *EGFR*-mutated cell lines was also inhibited at lower doses (HCC-827: IC_50_, 0.06 µM; SE, 0.005; PC-9: IC_50_, 0.04 µM; SE, 0.004), whereas no effect on the growth of the *BRAF* class 1–mutated cell line (HCC-364: IC_50_, >25 µM) and the *KRAS*-mutated cell line (ADK-17: IC_50_, >25) was observed (Fig 3C; Appendix Table A1). Notably, the growth of the *BRAF* class 2–mutated cell line was also inhibited at doses similar to those inhibiting *BRAF* class 3–mutated cell lines (NCI-H1395: IC_50_, 5.51 µM; SE, 1.60; Fig 3C; Appendix Table A1), consistent with previous findings on the direct inhibiting effect of EGFR-TKIs on the BRAF^G469^-mutated protein.^11^ Since 3D models may allow a better interpretation of TKI activity, erlotinib was tested also on 3D soft agar cultures and sphere formation assays.^12^ Consistent with previous observations, erlotinib reduced the 3D soft agar growth of the two *BRAF* class 3–mutated cell lines, PDX-ADK-36 (IC_50_, 0.23 µM; SE, 0.04) and ADK-14 (IC_50_, 1.01 µM; SE, 0.22), as well as that of the two *EGFR*-mutated cell lines (HCC-827: IC_50_, <0.01 µM; PC-9: IC_50_, 0.03 µM; SE, 0.02) and the *BRAF* class 2–mutated cell line (NCI-H1395: IC_50_, 0.05 µM; SE, 0.02). Instead, on comparison, erlotinib had a dismal effect on the growth of the *BRAF* class 1–mutated cell line (HCC-364: IC_50_, 5.81 µM; SE, 0.12) and the *KRAS*-mutated cell line (ADK-17: IC_50_, >10; Fig 3D). Similar findings were observed with sphere formation assays, as erlotinib exerted the strongest effect on *EGFR*-mutated cells (HCC-827: IC_50_, <0.01 µM; PC-9: IC_50_, 0.05 µM; SE, 0.01), followed by *BRAF* class 3–mutated cells (PDX-ADK-36: IC_50_, 0.11 µM; SE, 0.02; ADK-14: IC_50_, 0.34 µM; SE, 0.04) and *BRAF* class 2–mutated cells (NCI-H1395: IC_50_, 4.75 µM; SE, 1.63), whereas a remarkably weaker effect was observed in *BRAF* class 1– (HCC-364: IC_50_, 12.67 µM; SE, 0.86) and *KRAS*-mutated cells (ADK-17: IC_50_, 9.34 µM; SE, 0.46; Fig 3E).

### Activity of Other EGFR-Directed and Non-EGFR–Directed Agents in *BRAF* Class 3–Mutated NSCLC Cell Lines

**FIG A2.**
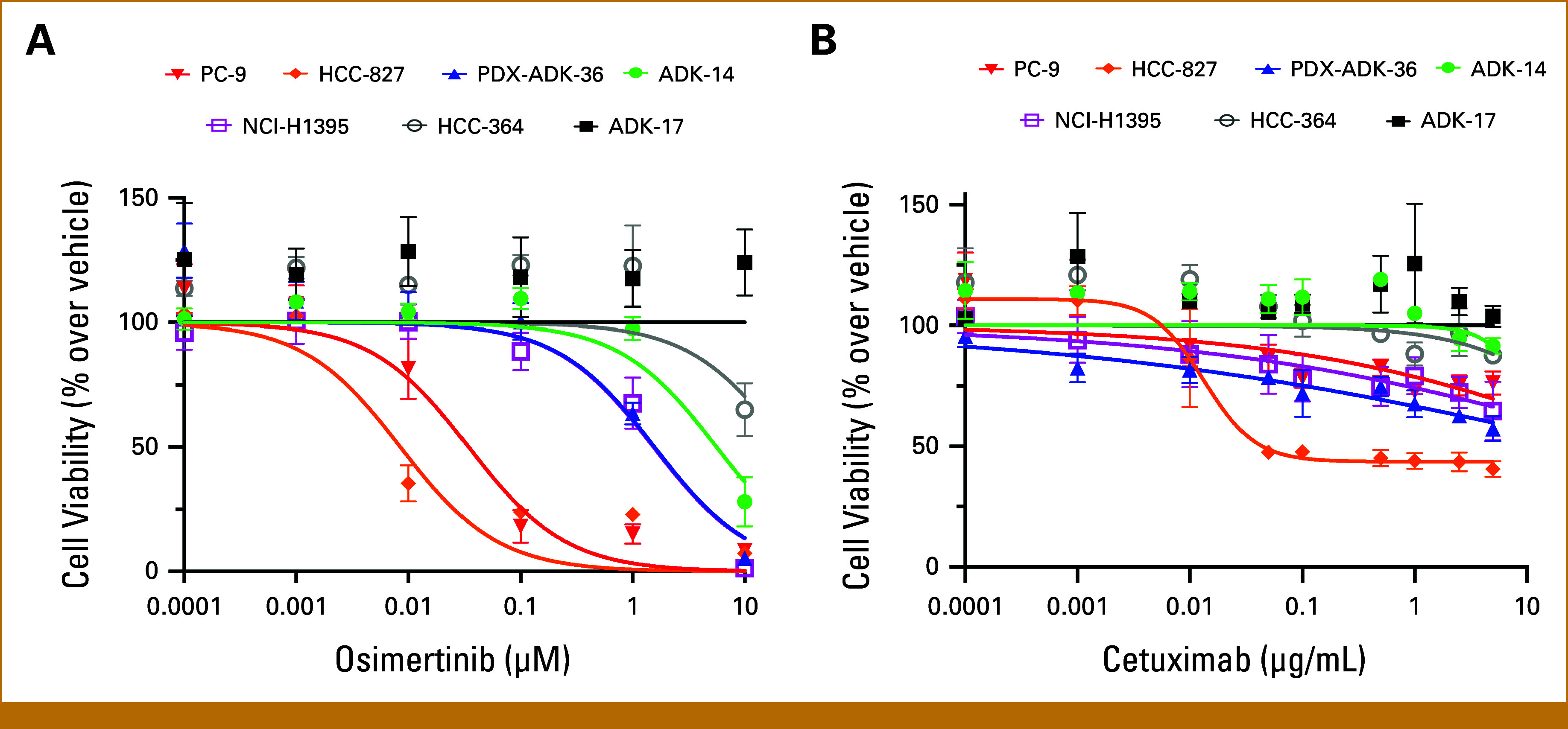
Comparative sensitivities to osimertinib and cetuximab. Effect of progressively increasing doses of (A) osimertinib (n = 2-6 experiments, each one with three replicates) and (B) cetuximab (n = 2-3 experiments, each one with three replicates) on cell 2D growth. 2D, 2-dimensional.

Following our observations, we explored whether the sensitivity of *BRAF* class 3–mutated NSCLC cell lines was limited to the first-generation EGFR-TKI or could be extended to osimertinib, a third-generation EGFR-TKI currently representing the standard of care for patients with *EGFR*-mutated NSCLCs. We observed that osimertinib inhibited the growth of the two *BRAF* class 3–mutated cell lines (PDX-ADK-36: IC_50_, 1.61 µM; SE, 0.32; ADK-14: IC_50_, 7.17 µM; SE, 2.20), although at higher doses compared with the two *EGFR*-mutated cell lines (HCC-827: IC_50_, 0.009 µM; SE, 0.003; PC-9: IC_50_, 0.04 µM; SE, 0.02; Appendix Fig A2A). Again, an inhibitory effect was also observed on the *BRAF* class 2–mutated cell line (NCI-H1395: IC_50_, 1.63 µM; SE, 0.58), whereas no effect was observed on the *BRAF* class 1–mutated cell line (HCC-364: IC_50_, >18 µM) or on the *KRAS*-mutated cell lines (ADK-17: IC_50_, >149 µM; Appendix Fig A2A). Sensitivities to the EGFR-directed monoclonal antibody cetuximab are shown in Appendix Figure A2B.

**FIG 4.**
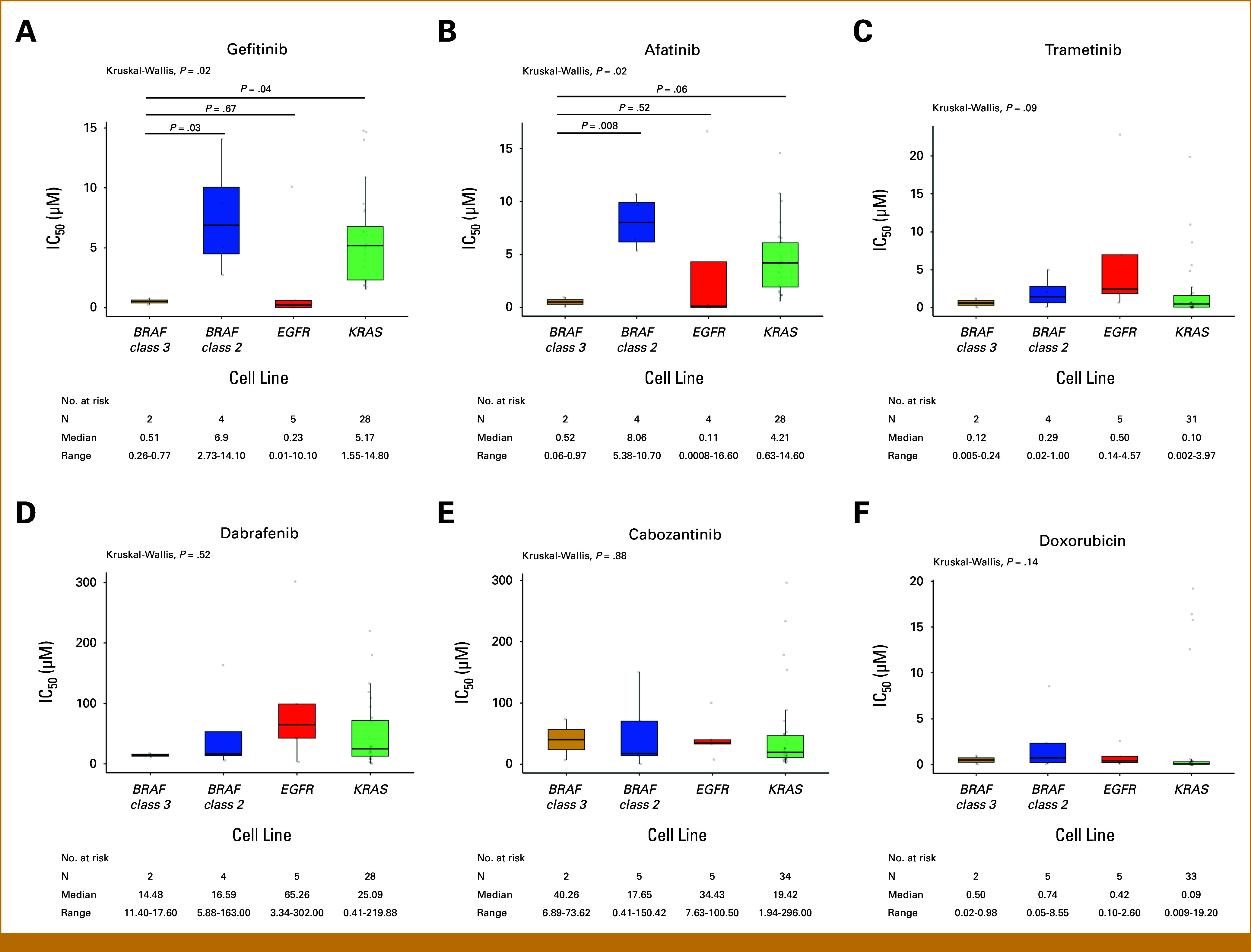
Activity of multiple agents in non–small-cell lung cancer cell lines with *BRAF* class 3, *BRAF* class 2, *EGFR*, or *KRAS* driver alterations in the Cell Line Encyclopedia. Activity, expressed as median IC_50_, of the EGFR inhibitors (A) gefitinib and (B) afatinib, (C) the MEK inhibitor trametinib, (D) the BRAF inhibitor dabrafenib, (E) the multityrosine kinase inhibitor cabozantinib, and (F) doxorubicin. IC, half maximal inhibitory concentration.

To provide an external validation for our findings, we interrogated the Cancer Cell Line Encyclopedia (Broad, 2019) via cBioPortal^13-16^ for NSCLC cell lines harboring *BRAF* class 3 mutations. Two cell lines of *BRAF* class 3–mutated NSCLC and available treatment data were identified, both with the BRAF^G466V^ class 3 mutation, which is identical to the mutation found in ADK-14, and without other concurrent driver alteration. In addition, cell lines of NSCLC harboring *BRAF* class 2 (N = 5), *EGFR* (N = 5), and *KRAS* (N = 36) mutations were identified and used as controls (Data Supplement). All included driver mutations were classified as oncogenic or likely oncogenic by OncoKB.^17^ *EGFR* mutations only included exon 19 deletions or L858R mutations, and cell lines harboring an *EGFR* T790M co-mutation were excluded given their known lack of sensitivity to first- and second-generation EGFR-TKIs. We explored the sensitivity of these cell lines to multiple agents, including EGFR-TKIs of first (gefitinib) and second generation (afatinib), a MEK inhibitor (trametinib), a BRAF inhibitor (dabrafenib), a multi-TKI (cabozantinib), and chemotherapy (doxorubicin; Data Supplement). Statistically significant differences in drug sensitivity among the four oncogene-addicted cell lines were only observed when exposed to the EGFR-TKIs gefitinib (*P* = .02) and afatinib (*P* = .02), mainly driven by their higher activity in *BRAF* class 3–mutated and *EGFR*-mutated cell lines compared with *BRAF* class 2–mutated and *KRAS*-mutated cell lines (Fig 4). Specifically, *BRAF* class 3–mutated cell lines exhibited a median IC_50_ of 0.51 µM (range, 0.26-0.77) when treated with gefitinib and 0.52 µM (range, 0.06-0.97) when treated with afatinib. These values were significantly lower than the median IC_50_ observed among *BRAF* class 2–mutated (gefitinib: 6.9 µM, *P* = .03; afatinib: 8.06 µM, *P* = .008) and *KRAS*-mutated cell lines (gefitinib: 5.17 µM, *P* = .04; afatinib: 4.21 µM, *P* = .06), but comparable with the IC_50_ displayed by *EGFR-mutated* cell lines (gefitinib: 0.23 µM, *P* = .67; afatinib: 0.11 µM, *P* = .52; Figs 4A and 4B). No differences in sensitivity to other agents were observed across cell lines (Figs 4C-4F).

## DISCUSSION

Patients with NSCLC harboring *BRAF*^non-V600^ alterations are a heterogeneous population in terms of clinicopathologic characteristics, genomic landscape, and BRAF kinase domain activity.^18^ These patients are currently orphans of targeted therapies and are treated as nononcogene-addicted, representing a relevant unmet clinical need. In this study, we demonstrate that *BRAF* class 3–mutated NSCLC may be targeted by EGFR-TKIs. Similar to the National Cancer Institute exceptional response initiative, our study started from a clinical retrospective observation: two patients with *EGFR* wild-type NSCLC who responded to erlotinib.^19^ Then, we established *BRAF* class 3–mutated NSCLC cell lines and confirmed their sensitivity to EGFR-TKIs. We further validated our findings using an independent, publicly available data set. In our experiments, a *BRAF* class 2–mutated cell line harboring the BRAF^G469A^ mutation used as one of the controls exhibited sensitivity to EGFR-TKIs, comparable with that observed in *BRAF* class 3–mutated cell lines. However, it did not show high levels of EGFR activation or a reduced pEGFR/EGFR ratio following erlotinib treatment. These observations align with those of a recent study indicating that NSCLC harboring a BRAF^G469V^ class 2 mutation may respond to EGFR-TKIs via direct binding to the mutant BRAF protein.^11^ However, this mechanism appears unlikely to apply to *BRAF* class 3 mutations, given the absence of intrinsic BRAF kinase activity characterizing them, the elevated EGFR activation found in cells harboring these mutations, and its reduction under erlotinib treatment.^5^ Therefore, we hypothesize that in *BRAF* class 3–mutated NSCLC, the mutant BRAF protein amplifies a RAS signal already triggered upstream by a hyperactivated wild-type EGFR, a signal insufficient on its own to drive cancer proliferation without the *BRAF* mutation. Hyperphosphorylation of the EGFR receptor has been previously reported in *BRAF* class 3–mutated NSCLC and colorectal cancer (CRC) cells, but not in malignant melanoma cells.^6^ Consistently, EGFR inhibition with erlotinib or cetuximab was effective in *BRAF* class 3–mutated NSCLC and CRC cell lines. Moreover, anti-EGFR antibodies have demonstrated high activity in patients with metastatic *BRAF* class 3–mutated CRC while showing low activity in those harboring class 2 mutations.^6,20^ Notably, other potential targets reported to be active in some cases of *BRAF* class 3–mutated tumors, such as MET, and erlotinib off-target effects that may contribute to cell growth inhibition were not explored in this study and may be object of further investigation in future research.^6^

To our knowledge, this is the first report of the clinical activity of EGFR inhibition in patients with *BRAF* class 3–mutated NSCLC. The main limitation of this study is the availability of only two patients treated with erlotinib, reflecting the low prevalence of *BRAF* class 3 mutations in NSCLC (approximately 1%) and the historically limited use of erlotinib in later lines of treatment for *EGFR* wild-type patients. Nevertheless, the strengths of this study include the consistency between clinical and preclinical data and the reproducibility of our findings in an independent data set.

In conclusion, the activation of wild-type EGFR may play a significant role in *BRAF* class 3–mutated NSCLC, which currently represents a population orphan of targeted therapies, suggesting that these tumors might be responsive to EGFR-TKIs. These findings warrant validation through prospective clinical studies, as *BRAF* class 3 mutations might identify an additional subset of patients with NSCLC who could benefit from existing targeted therapies.

## APPENDIX

**TABLE A1.** IC_50_ (µM) Values for Each Experiment in Cell Lines Tested for Erlotinib 2D Growth Inhibition

Experiment Number	Cell Line						
	PC-9	HCC-827	PDX-ADK-36	ADK-14	NCI-H1395	HCC-364	ADK17
1	0.04099	0.06233	9.232	5.597	3.903	25.56	8.15E + 40
2	0.02704	0.05408	10.07	6.183	7.108	31.93	1.28E + 33
3	0.03751	0.07072	5.207	8.695	—	48.88	25.84
4	—	—	0.806	7.963	—	—	81.84
5	—	—	—	—	—	—	34.83

Abbreviations: 2D, 2-dimensional; IC, half maximal inhibitory concentration; PDX, patient-derived xenograft.
